# Doctors Who Advertise

**Published:** 1890-08

**Authors:** 


### DOCTORS WHO ADVERTISE.

The code of ethics of the American Medical Association is a deliberate expression of the views of the profession upon the fundamental principles underlying the practice of medicine. It is the court of last resort upon all questions of an ethical nature, and from its verdict there is no appeal. The principles therein expressed are not merely a set of arbitrary rules agreed upon for the common good, but there is a moral principle underlying them, which make them a code of *ethics* in the true sense of that word.

The code condemns advertising by physicians in unequivocal terms. Its condemnation is based upon the theory that to advertise professional skill is to vaunt one's own personal qualifications, and to boast of merits which are better judged by others than by one's self. Such a course of action is repugnant to all right-minded men, for the same reason that they would hesitate to boast of their bravery, honesty, or other personal characteristics for the sake of gain. The feeling is so instinctive to every sensitive and refined nature that it scarcely admits of or requires definition.

There are three classes of doctors who advertise. The first is the avowed charlatan, who regards the practice of medicine as a mere means of making money, who parades his own skill before the public as the merchant extols the quality of his wares; who advertises openly and systematically, and who subscribes to no code but that of money-making. Such men are the pariahs of the profession, to whom it would be as useless to preach moral principle as it would be to attempt to instil the quality of mercy into a royal Bengal tiger.

The second class is made up of men who, by reason of their obscurity or lack of success in practice, are constantly subject to financial stress, and who, while recognizing the moral principle involved, are constrained by the exigencies of poverty to do that which their consciences condemn. They surreptitiously avail themselves of opportunities to get their names before the public, in hope of thereby increasing their practices and augmenting their scanty incomes. We feel sorry for such men, and are inclined to excuse their violations of the code on the ground that "necessity knows no law." But they labor under a delusion. The public is not deceived. The motive is so palpable that he who runs may read it, and the result is to defeat the very end in view.

The third class of advertisers is made up of men high in their profession, who have achieved eminence, who love notoriety for notoriety's sake, and who are willing to purchase it at the price of principle; men who are "ever haunted by the dreadful secret of their own pre-eminence," and who fear that the public eye may sometimes wander for a moment to fix its gaze upon some lesser light. Specialists, who have achieved wealth and renown, are often found in this class, and we find their pictures smiling at us from the pages of secular journals, accompanied by fulsome accounts of their "valuable services to the public." Such men tell us that these things are done without their knowledge or consent, and are inevitable results of their high reputation. But they fail to explain why other men of equal reputation are never made the victims of such accidents.

Such practices are utterly reprehensible and morally wrong. They degrade the noble calling of medicine, and furnish an excuse for imitation to humbler members of the profession. An exalted position entails increased rather than diminished responsibilities, and he who would shield himself behind a high reputation to violate professional ethics is unworthy of the reputation which he possesses. There is no middle ground in this matter. The true physician must, like Cæsar's wife, be above suspicion.

The JOURNAL has always fought this evil, and will in the future, as in the past, attack it whenever it rears its head. If any man should feel aggrieved by the expression of our views, we would simply point him to the code of ethics, to whose principles he has voluntarily subscribed, and say, "We are but seconding your condemnation, which there lies recorded."

